# Comparison of therapeutic efficacy and treatment costs of self-expandable metal stents and plastic stents for management of malignant biliary obstruction

**DOI:** 10.1186/s12876-023-02668-9

**Published:** 2023-02-16

**Authors:** Renáta Bor, Anna Fábián, Mónika Szűcs, Anita Bálint, Mariann Rutka, Tibor Tóth, László Czakó, Klaudia Farkas, Norbert Buzás, Ágnes Milassin, Tamás Molnár, Zoltán Szepes

**Affiliations:** 1 First Department of Medicine, University of Szeged, Kálvária Sgt 57, Szeged, 6725 Hungary; 2 Department of Medical Physics and Informatics, University of Szeged, Szeged, Hungary; 3 Department of Health Economics, Faculty of Medicine, University of Szeged, Szeged, Hungary

**Keywords:** Malignant biliary obstruction, SEMS, Plastic stent, Cost-effectiveness

## Abstract

**Background:**

According to the European Society of Gastrointestinal Endoscopy guidelines, self-expandable metal stents (SEMSs) are preferable to plastic stents (PSs) in the management of pancreatic cancer, regardless of cancer stage. The aim of this study was to compare the therapeutic efficacy and treatment costs of SEMS and PS in the management of malignant biliary obstruction.

**Methods:**

One hundred and thirty-five patients who underwent endoscopic stent placement were retrospectively enrolled and divided into PS (41 patients), primary SEMS (39 patients) and secondary SEMS (55 patients) groups. We determined the technical and functional success rate, stent patency, and cumulative treatment cost.

**Results:**

A total of 111 SEMSs and 153 PSs were placed with similar technical (100% vs. 98.69%) and functional success rate (90.10% vs. 86.27%) but with different stent patency (10.28 vs. 22.16 weeks; *p* < 0.001). Multiple PS implantations and larger stent diameter increased the length of stent patency compared to 7-Fr PSs (10.88 vs. 10.55 vs. 7.63 weeks, respectively). The cumulative treatment cost of patients with different survival times did not differ significantly between groups, however, among patients surviving 2–4 months it was higher in PS group than primary SEMS and secondary SEMS groups (2888€ vs. 2258€ vs. 2144€, respectively, *p* = 0.3369) due to increased number of biliary reintervention (2.08 ± 1.04 vs. 1.20 ± 0.42 vs. 1.50 ± 0.53; *p* < 0.0274) and longer hospital stay (15.77 ± 10.14 vs. 8.70 ± 7.70 vs. 8.50 ± 6.17 days, *p* = 0.0527).

**Conclusions:**

In view of treatment costs, the consequences of illness, and the processes of the health care system, SEMS implantation is recommended regardless of patients’ life expectancy.

**Supplementary Information:**

The online version contains supplementary material available at 10.1186/s12876-023-02668-9.

## Background

Pancreatobiliary malignancies are often diagnosed at a locally advanced or metastatic stage, when curative resection is no longer feasible. These tumors' prognosis is especially dismal, and their 5-years mortality rate reaches 94% [1]. In approximately 70% of cases, some degree of biliary obstruction has already occurred at the time of diagnosis, regardless of stage, and such obstruction is frequently associated with decreased length of survival [2, 3]. Endoscopic retrograde cholangiopancreatography (ERCP) with placement of plastic stents (PS) or self-expandable metal stents (SEMS) is the first-choice procedure for the palliation of malignant obstruction of the infrahilar common bile duct (CBD). In the traditional approach, the choice of stent depends on the patient’s clinical condition and the disease stage. The most important advantages of PS over SEMS are the favorable upfront cost and the longer-term experience of healthcare staff in their usage; nevertheless, PS need to be replaced every 3 to 4 months to prevent or manage the complications, such as occlusion and migration. Longer stent patency of SEMS might compensate for its substantially higher cost. In guidelines published in 2012, the European Society of Gastrointestinal Endoscopy (ESGE) recommended the use of 10-Fr PS if the diagnosis of malignancy is not established or if the patient's life expectancy is less than 4 months [4]. In contrast, the newer guidelines, published in 2017, highlight the priority of SEMS usage, regardless of cancer stage [5] .

Although several international gastroenterological societies recommend the placement of SEMS with ERCP for the management of malignant biliary obstruction, the use of PS has not yet substantially decreased [6, 7].Therefore, we wished to determine whether PS have any advantage over SEMS in the daily clinical practice. In this retrospective study, we compared the therapeutic efficacy and cost effectiveness of SEMS with those of PS in the treatment of primary malignant biliary obstruction. During the analysis, we determined the technical and functional success rate of the stents, the duration of stent patency and the cumulative treatment costs.

## Methods

### Patient enrollment

We gathered retrospective data about consecutive patients with unresectable primary pancreatobiliary malignancy who underwent endoscopic stent placement for distal biliary obstruction in one of the Hungarian tertiary-level referral gastroenterology centers between 2011 and 2018. Biliary obstruction had been treated before the ESGE guideline updated in 2017 was published and integrated into daily routine. The interventions were performed by one of the three ERCP specialists in our institution. The exclusion criteria were (1) benign lesion such as chronic or autoimmune pancreatitis, identified by clinical or histological examination against the background of suspected malignant biliary obstruction; (2) surgical resection or biliary bypass performed less than 4 weeks after the first biliary stent implantation; (3) concurrent gastric outlet obstruction and malignant biliary obstruction at the time of stent placement; (4) hilar or intrahepatic malignant biliary obstruction; (5) secondary malignant biliary obstruction caused by disseminated extra-pancreatobiliary malignancy with direct tumor spread or lymph node metastasis; (6) moribund condition of patients with end-stage pancreatobiliary malignancy, which could be expressible numerically (5 or 6 points) as the score on the physical status classification of the American Society of Anesthesiologists (ASA; Additional file 1: Table S1). The study was approved by the Regional and Institutional Human Medical Biological Research Ethics Committee of the University of Szeged, Hungary (ethics approval number: 3680/2015 SZTE). The study was carried out in accordance with the Declaration of Helsinki.

### Determination of groups

During the study period, according to the existing guidelines, PSs were used in cases of potentially resectable pancreatobiliary adenocarcinomas or when the diagnosis and stage of malignancy were not clearly established. Primary SEMS placement was performed only for potentially unresectable disease, when the severe comorbidity or poor general condition of the patient precluded surgical resection, or when the patient did not consent to surgery. After the definitive diagnosis of unresectable pancreatobiliary malignancy, a previously inserted PS could be replaced with a SEMS (secondary SEMS [sSEMS]). This scheduled stent replacement was carried out 3 to 4 months after the PS implantation or earlier if stent complications (such as cholangitis, stent occlusion, or stent migration) occurred. Patients were accordingly divided into groups (PS, primary SEMS [pSEMS], and sSEMS) during the assessment of cost effectiveness of different stent types. We used 7-Fr and 10-Fr polyethylene straight PSs provided by Blue Neem Medical Devices Private Limited (Karnataka, India) and CONMED Corporation (Utica, NY, USA,) and 10-mm covered, partially covered, or uncovered biliary SEMSs with a diameter of 10 mm and a length of 40–80 mm provided by Changzhou Health Microport Medical Device Co., Ltd. (Changzhou, Jiangsu, China), Boston Scientific Corporation (Minneapolis, MN, USA); S&G BioTech Inc. (EGIS™; Yongin-si, Korea); ENDO-FLEX GmbH. (Voerde, Germany); Endo-Technik (Solingen, Germany); and Taewoong Medical Co., Ltd., (Gyeonggi-do, South Korea).

### Endpoints of the study

The primary endpoint of the study was to determine and compare the efficacy and treatment costs of PS and SEMS placement in the management of primary distal malignant biliary obstruction. The efficacy of stent implantation was characterized by technical and functional success rates and duration of stent patency. The intervention was considered technically successful if the stent was placed across the stricture in the proper position, as confirmed by radiography and endoscopy. Functional success of the stent was defined as restoration of bile outflow, detected by endoscopy immediately after drainage, and as more than 20% decrease in serum bilirubin level from baseline within a week after stenting. Duration of stent patency was defined as the period between the stent placement and either functional failure or the patient's death. The following complications were investigated: pancreatitis, cholangitis, stent occlusion, cholecystitis, bleeding, perforation. Complication rate was given as the proportion of patients with one or more adverse event. In addition, we determined also the rate of immediate procedure related and disease progression related complications, as well as the rate of early complications (occurred ≤ 4 weeks after stenting) causing stent insufficiency. Reintervention rate was defined as the proportion of patients who required endoscopic, interventional radiological or surgical intervention to ensure bile flow.

Length of patient survival time was highly variable in the three groups; therefore, the average treatment costs per month of survival were compared in the cost analysis. In the PS and pSEMS groups, the cost analysis evaluated the cost of the entire follow-up period, while in the sSEMS group we assessed both the entire follow-up period of patients (pre-SEMS and post-SEMS together) and the post-SEMS costs alone in order to determine the cost-effectiveness of switch from a PS to SEMS in comparison to definitive PS or SEMS placement. In addition, we compared the commutative treatment costs of patients with different survival times. In the sSEMS group, only the costs of the post-SEMS period were evaluated to determine whether the cost of switching to SEMS was recovered. During the economic analysis, we assessed only the costs of medical treatment directly associated with the management of biliary obstruction: the cost of stents (PS: 32€; SEMS: 540€), interventions (ERCP: 320€; endoscopic sphincterotomy: 95€; percutaneous transhepatic drainage with plastic stent: 350€; percutaneous transhepatic drainage with metal stent: 910€), and hospital stay (including the laboratory tests, antibiotics, medicines, infusions, and nursing: 130€/day; Additional file 1: Table S2).

### Statistical analysis

To collect the medical documentation of patients, we used a MedSolutions medical recorder. Statistical analysis was performed with SPSS software version 24 (SPSS Inc., Chicago, IL, USA); *p* values of less than 0.05 were considered significant. Descriptive statistics were expressed as means and medians with ranges. Differences in continuous variables such as survival time and duration of stent patency were assessed with an independent samples *t* test. Chi-square and Fisher’s exact tests were used to compare the proportion of categorical variables such as technical and functional success and ASA scores. The differences in cost-effectiveness between the PS, pSEMS, and sSEMS groups were assessed with ANOVA technique. We used logistic regression analysis, Fisher’s exact test, and chi-square test to identify the factors that could modify the cost-effectiveness of stenting.

## Results

### Demographic and clinical data of patients

Of the 135 patients with primary malignant biliary obstruction, 41 were in the PS group, 39 were in the pSEMS group, and 55 were in the sSEMS group. The clinical characteristics of patients (gender, age, ASA score) and neoplasms (type of primary tumor, location of obstruction, rate of distant metastasis, and use of chemoradiotherapy) did not differ significantly. Mean survival was substantially longer among patients in the sSEMS group (47.07 ± 32.79 weeks) than among those in the pSEMS (21.46 ± 20.87 weeks) and PS groups (18.26 ± 16.53 weeks; *p* < 0.001; Table 1). All neoplasms involved only the CBD without the infiltration of hilar part, with the obstruction of ampullary and distal part of CBD being the most frequent (77.04%).

**Table 1:** Clinical and demographic data of enrolled patients

Characteristic	Plastic stent (n = 41)	Primary SEMS (n = 39)	Secondary SEMS (n = 55)	*p* Value
Female/male (Nr)	30-Nov	22/17	29/26	0.1116
Age (years)	70.27 ± 13.19	73.79 ± 13.31	71.82 ± 9.07	0.406
Obstruction location				0.4683
- Ampullary	4 (9.76%)	2 (5.13%)	4 (7.27%)	
- Distal CBD	29 (70.73%)	34 (87.18%)	41 (74.55%)	
- Proximal CBD	8 (19.51%)	3 (7.69%)	10 (18.18%)	
*Type of primary tumor*				
- Pancreatic adenocarcinoma	28 (68.29%)	35 (89.74%)	37 (67.27%)	0.5674
- Cholangiocarcinoma	6 (14.63%)	1 (2.56%)	9 (16.36%)	
- Ampullary cancer	4 (9.76%)	3 (7.69%)	4 (7.27%)	
- Gallbladder carcinoma	3 (7.32%)	0 (0%)	5 (9.09%)	
- Distant metastasis	23 (56.10%)	19 (48.72%)	25 (45.45%)	0.6273
Chemotherapy or radiation (Nr)	13 (31.71%)	17 (43.59%)	31 (56.36%)	0.0544
*ASA score*				
- ASA I	7 (17.07%)	13 (33.33%)	24 (43.64%)	0.0681
- ASA II	20 (48.78%)	12 (30.72%)	17 (30.09%)	
- ASA III	14 (34.15%)	14 (35.89%)	14 (25.45%)	
Survival (weeks)	18.26 ± 16.53	21.46 ± 20.87	Pre- and post-SEMS period:	< 0.001
			63.61 ± 44.39	
			Post-SEMS period:	
			47.07 ± 32.79	

*ASA* American Society of Anesthesiologists; *CBD* common bile duct

### Efficacy of PSs and SEMSs

The 135 participants underwent ERCP with placement of 264 stents, of which 111 were SEMSs and 153 were PSs (Table 2). The different stent types were not evenly distributed in the groups: covered stents (57.66%) were the most frequently used SEMS, and in the PS group, the proportion of patients receiving 10-Fr stents (47.06%) was almost equal to the proportion receiving simultaneous multiple stent placements (39.22%). The efficacy of stents was assessed independently of the cost-effectiveness group in which stenting was performed. In 135 of 264 cases, the efficacy analysis affected the first biliary stent implantation of patient. At least one stent implantation had previously done in 37.25% of PSs and 64.86% of SEMSs (SEMS implantation after removal of PS N = 55; SEMS in SEMS N = 17).

**Table 2:** Comparison of effectiveness of plastic stent and self-expandable metal stent placement in the management of malignant biliary obstruction

Effectiveness of stent placement			
	Plastic stent (n = 153)	SEMS (n = 111)	*p* Value
Technical success rate	98.79%	100%	0.5108
Functional success rate	86.27%	90.09%	0.4553
Mean stent patency	10.28 ± 10.02	22.16 ± 22.23	< 0.001

Effectiveness of stent type					
Plastic stent		*p* Value	SEMS		*p* Value
*Technical success rate*					
7-Fr (n = 21)	100%	0.6285	Covered (n = 64)	100%	0.9999
10-Fr (n = 72)	97.22%		Partially covered (n = 10)	100%	
Multiple stenting (n = 60)					
	100%		Uncovered (n = 37)	100%	
*Functional success rate*					
7-Fr (n = 21)	85.71%	0.5519	Covered (n = 64)	85.59%	0.2898
10-Fr (n = 72)	83.33%		Partially covered (n = 10)	100%	
Multiple stenting (n = 60)	90.00%				
			uncovered (n = 37)	94.59%	
*Mean stent patency*					
7-Fr (n = 21)	7.06	0.422	covered (n = 64)	21.35	0.9999
10-Fr (N = 72)	10.56		partially covered (n = 10)	22.53	
Multiple stenting (n = 60)					
	10.88		uncovered (n = 37)	22.8	

*SEMS* self-expandable metal stent

The rates of technical success (100% vs. 98.69%) and functional success (90.10% vs. 86.27%) of SEMSs and PSs were similarly high and independent of stent type. The mean duration of patency of SEMSs was significantly longer compared with PS (22.16 vs. 10.28 weeks; *p* < 0.001). Stent failure developed significantly later in cases of younger patients who had only mild comorbidities (ASA I: 29.4 weeks; ASA II: 17.0 weeks; ASA III: 18.2 weeks; *p* = 0.0210), and a weak correlation was observed between stent patency and location of obstruction in the SEMS group (ampullary location: 48.4 weeks; distal location: 21.8 weeks; proximal location: 12.8 weeks; *p* = 0.0066). These factors did not influence the therapeutic effect of PSs; however, duration of stent patency was increased by implantation of multiple stents (10.88 weeks) and larger stent diameter (10.55 weeks) in comparison with the use of single 7-Fr stents (7.63 weeks), although the difference was not significant (*p* = 0.4420).

The rate of overall complications of PSs was significantly higher than that of SEMS (74.51% vs. 48.65%, respectively; *p* < 0.0512), but the immediate procedure related complications did not differ between groups (2.6% vs. 2.7%). Stent occlusion necessitated the replacement of PSs in 68.05% of cases, and concurrent cholangitis was observed in 64.05% of cases. Total 29 patients with PS (18.95%) had early complications (stent occlusion N = 25; stent migration N = 2; cholangitis N = 24) causing stent insufficiency and requiring biliary reintervention, which was substantially more frequent than for SEMS (6.31%; cholangitis N = 5; stent migration N = 2; early tumor ingrowth N = 3). No substantial difference was detectable among covered, partially covered and uncovered SEMSs in terms of overall stent complications (46.88% vs. 60.00% vs. 48.65%, respectively), tumor ingrowth (37.50% vs. 40.00% vs. 35.14%, respectively), cholangitis (35.94% vs. 50.00% vs. 43.24%, respectively), cholecystitis (3.12% vs. 10.00% vs. 2.70%, respectively), or stent migration (3.12% vs. 0.00% vs. 2.70%, respectively).

### Cost analysis of PSs and SEMSs

**Fig. 1:**
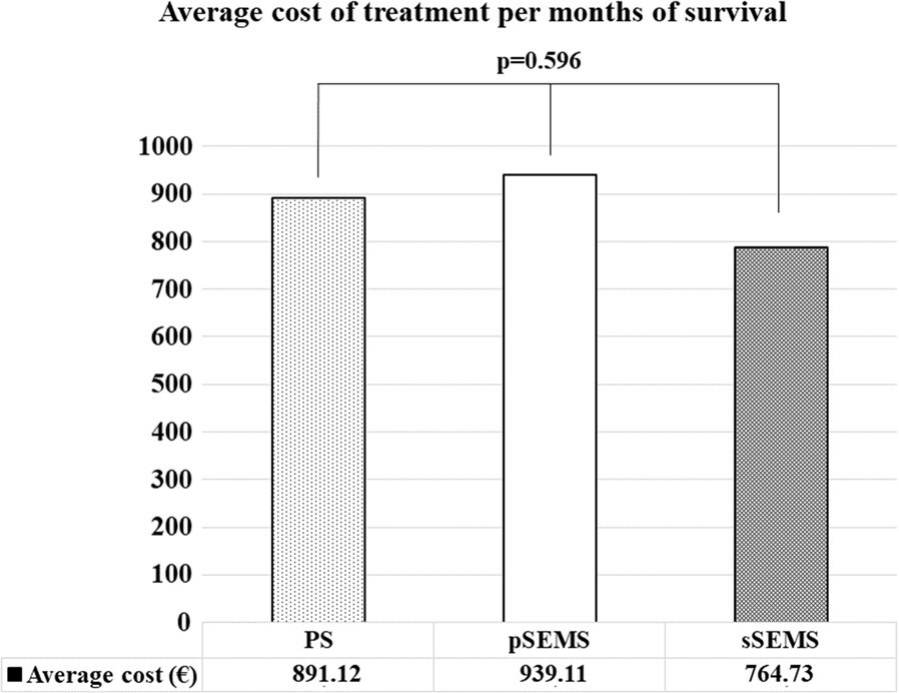
Costs of treatment per months of survival were not significantly different among patients receiving the plastic stent (PS), primary self-expandable metal stent (pSEMS), and secondary self-expandable metal stent (sSEMS)

**Fig. 2:**
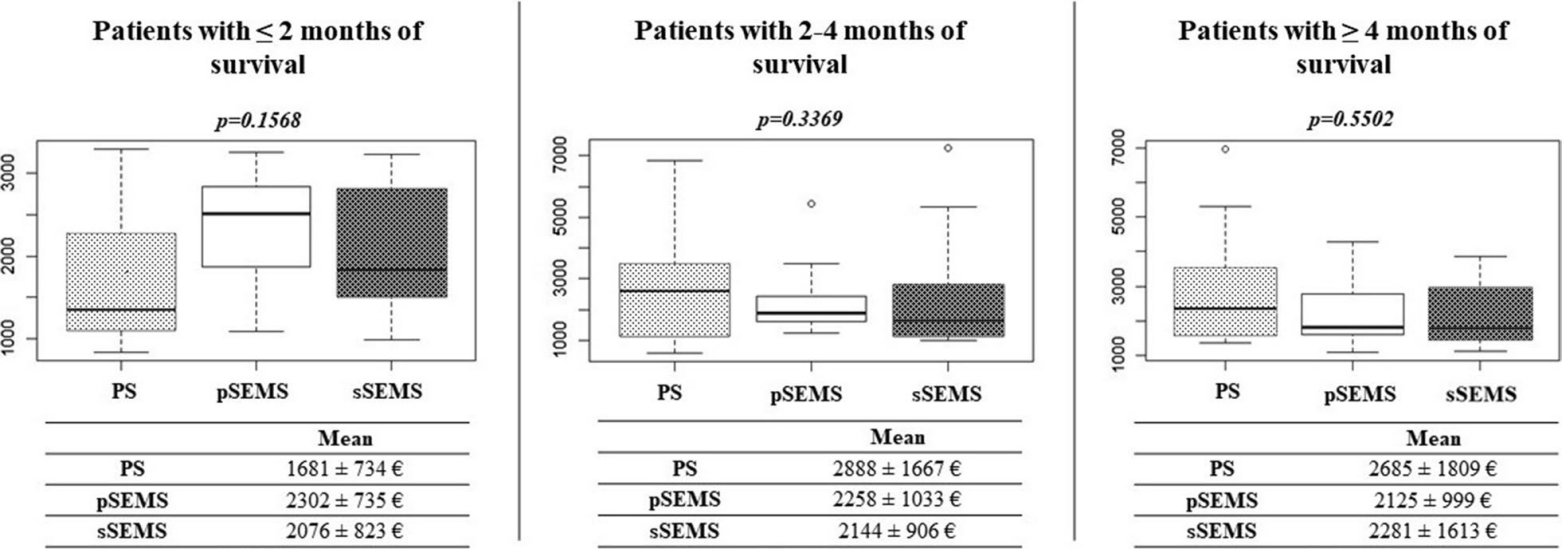
Length of survival did not influence the cost effectiveness of stent placement. PS, plastic stent; SEMS, self-expandable metal stent; pSEMS, primary SEMS; sSEMS, secondary SEMS

There was no difference in the average cost of treatment per month among the PS (891.12€), pSEMS (939.11€), and sSEMS groups assessed the whole follow-up period (764.73€; *p* = 0.596; Fig. 1). No significant difference was observed when only the post-SEMS period of the sSEMS group (788.45€; *p* = 0.784) was compared with the other two groups. We compared the cumulative treatment costs for patients with different survival times. In the PS group, 31.71% of patients required repeated ERCP and PS implantation due to complications before the planned stent replacement date. Among patients with short survival (≤ 2 months), the cumulative treatment costs did not differ significantly by stent groups: 1681 ± 734€ for PSs, 2302 ± 735€ for pSEMSs, and 207 ± 823€ for sSEMSs (*p* = 0.1568). Among patients who survived 2 to 4 months, repeated biliary intervention was performed in substantially more PS recipients than pSEMS and sSEMS recipients (2.08 ± 1.04 vs. 1.20 ± 0.42 vs. 1.50 ± 0.53; p < 0.0274), and this trend was also observed among patients who survived more than 4 months (PS: 2.20 ± 1.15; pSEMS: 1.26 ± 0.56; sSEMS: 1.84 ± 1.51; *p* < 0.0812). The PS implantation was also associated with longer hospitalization among patients surviving longer than 2 months. (Table 3) Therefore, among patients surviving 2 to 4 months, the cumulative cost of treatment was higher for PSs than for pSEMS and sSEMS (2888€ vs. 2258€ vs. 2144€, respectively, p = 0.3369), and this trend was the same among patients surviving 4 months or longer (2685€ vs. 2125€ vs. 2281€, respectively; *p* = 0.5502), but the differences were not statistically significant (Fig. 2).

**Table 3:** Comparison of plastic stent, primary SEMS, and secondary SEMS in terms of biliary reintervention rate, length of hospital stays, and number of repeated hospitalizations

Survival time	PS	pSEMS	sSEMS	*p* Value
*Biliary reintervention (number)*				
≤ 2 months	1.23 ± 0.60	1.30 ± 0.48	1.29 ± 0.47	0.9423
2–4 months	2.08 ± 1.04	1.20 ± 0.48	1.50 ± 0.53	0.0274
≥ 4 months	2.20 ± 1.15	1.26 ± 0.56	1.84 ± 1.51	0.0812
*Length of hospital stay (days)*				
≤ 2 months	8.92 ± 4.80	9.60 ± 4.80	8.14 ± 5.16	0.7756
2–4 months	15.77 ± 10.14	8.70 ± 5.70	8.50 ± 6.17	0.0527
≥ 4 months	12.67 ± 9.72	8.11 ± 5.71	7.11 ± 7.23	0.06415
*Repeated hospitalization (number)*				
≤ 2 months	1.38 ± 0.65	1.10 ± 0.32	1.14 ± 0.36	0.2911
2–4 months	2.23 ± 1.09	1.30 ± 0.48	1.30 ± 0.48	0.009
≥ 4 months	2.13 ± 1.06	1.32 ± 0.58	1.68 ± 1.22	0.0818

*SEMS* self-expandable metal stent

## Discussion

Malignant biliary strictures and painless obstructive jaundice are most commonly caused by either pancreatic cancer or cholangiocarcinoma, both of which are often diagnosed at a locally advanced stage or when distant metastasis has already occurred.[6]The rate of 5-year survival with both cancers at this advanced stage is very poor: only 1% to 5% [1, 8, 9]. Palliative biliary drainage should be performed for all patients with unresectable disease and before neoadjuvant chemotherapy; furthermore, biliary stenting is required in resectable cases complicated with cholangitis or severe symptomatic jaundice, or if surgery is delayed [5, 10]. Our study showed that the duration of patency of SEMSs (22.16 weeks) was almost twice that of PSs (10.28 weeks; p < 0.001), which is consistent with the results of previously published clinical trials [11, 12].

In a 2016 meta-analysis, Moole et al. evaluated the data of 984 patients from four retrospective and seven randomized controlled trials and demonstrated that duration of patency of SEMSs (median, 167.7 days; 95% confidence interval [CI], 159.2 to 176.3) was superior to that of PS (median, 73.3 days; 95% CI, 69.8 to 76.9), and that SEMSs had lower rates of occlusion (odds ratio [OR], 0.48; 95% CI, 0.34 to 0.67) and reintervention (OR, 1.1; 95% CI, 0.9 to 1.3) than did PSs (OR, 1.7; 95% CI, 1.5 to 1.9) [13]. Pooled analysis of randomized controlled trials did not reveal differences between PS and SEMS in overall patient survival (weighted mean difference, 0.67 months; 95% CI, $-$ 0.66 to 1.99) or in the 30-day mortality odds ratio (0.80; 95% CI, 0.52 to 1.24), but the rate of symptom-free survival at 6 months was higher (OR, 5.96; 95% CI, 1.71 to 20.81]) [14].

The early clinical trials and meta-analyses suggested that SEMS placement is the right choice for cost-effectiveness considerations only if a patient’s life expectancy is more than 4 months [15–17]. According to the previous ESGE guideline published in 2012, the initial insertion of a 10-Fr PSs was recommended if the diagnosis of malignancy was not established or if expected survival was shorter than 4 months [4]. In contrast, more recent trials have demonstrated that the total cost of PS and SEMS per patient did not differ among patients with short (3-month) survival or metastatic disease despite the fact that SEMS placement was initially more expensive [18]. Furthermore, the general and disease-specific health-related quality of life of patients with inoperable malignant extrahepatic bile duct obstruction was better over time with SEMSs than with PSs [19]. In addition, a German retrospective study of the management of SEMS occlusion did not reveal significant differences in median overall duration of secondary stent patency (88 days for sSEMS, 143 days for PS; *p* = 0.069), median subsequent intervention rate (53.4% for sSEMS, 40.0% for PS; *p* = 0.501), or median case costs (5145€ for sSEMS, 3473€ for PS; *p* = 0.803) [20].

In view of new evidence, the ESGE (in the guideline published in 2017) now recommends SEMS insertion for palliative drainage of malignant extrahepatic biliary obstruction, regardless of the patient's life expectancy [5]. The results of our study confirmed that use of PS is not superior to that of SEMS with regard to the cumulative cost of treatment even in cases of short (≤ 2 month) survival, but the total hospitalization time is longer, and the reintervention rate is higher.

The most appropriate SEMS type in the management of malignant distal biliary obstruction is still debated. Meta-analyses have revealed no significant difference between covered and uncovered metal stents with regard to the survival benefit, overall rate of adverse events, rate of stent dysfunction, and duration of primary stent patency during the period from primary stent insertion to primary stent dysfunction or patient death [21–23]. Some studies, however, have suggested that the covered SEMS is associated with a lower risk of tumor ingrowth but higher risks of tumor overgrowth, sludge formation, stent migration, and post-stenting cholecystitis [24–26]. In our cohort, the coverage of stent did not influence the technical and functional success rate, stent patency, complication rate, or cost-effectiveness of stenting.

The main limitation of this research is its retrospective, single-center design. Thus, some differences were observed in terms of stent choice and timing of stent replacement. The designs of SEMS purchased from different manufacturers varied slightly, and the diameter and the number of PS inserted in the same time were different, but their design was uniform. We considered these differences during the statistical analysis, but the substantial difference in the sizes of group populations limited the detection of statistically significant variance. Because of the retrospective nature of data collection, the only detailed information available concerned the gastroenterological treatment of pancreatobiliary malignancies performed in our tertiary-level clinical center; however, the patients frequently underwent follow-up in primary- or secondary-level medical institutions. Therefore, in the cost-effectiveness analysis, we assessed the direct cost of interventions and hospitalization in relation to malignant biliary obstruction. The concomitant oncologic treatments or coexisting diseases with potential influence on the total cost of patients' medical care would not be included in the analysis. The ASA score represented the clinical condition of patients.

## Conclusion

Our retrospective cohort study confirmed that SEMS is a better choice than PS in the management of unresectable primary malignant biliary obstruction not only in terms of effectiveness and longer stent patency but also in terms of cost-effectiveness. Because we found no difference in the cumulative treatment costs of patients with different survival times, we recommend SEMS implantation regardless of patients’ life expectancy. Our results also confirmed that multiple stent implantation and larger stent diameter increased the duration of stent patency and decreased the reintervention rate, in comparison with the use of single 7-Fr stents. Therefore, if SEMS is not available, implantation of multiple PSs is recommended.

## Supplementary Information

**Additional file 1**: **Table S1**. American Society of Anesthesiologists (ASA) Physical Status Classification; **Table S2**. Costs used in cost-effectiveness analysis of stent implantation in the management of primary malignant biliary obstruction.

## Data Availability

The datasets used and/or analyzed during the current study are available from the corresponding author on reasonable request.

## Abbreviations

ASA: American Society of Anesthesiologists
CBD: Common bile duct
ERCP: Endoscopic retrograde cholangiopancreatography
ESGE: European Society of Gastrointestinal Endoscopy
SEMS: Self-expandable metal stent
pSEMS: Primary SEMS
PS: Plastic stent
PTD: Percutaneous transhepatic drainage
sSEMS: Secondary SEMS
