# Supplementary material for: Comparison of therapeutic efficacy and treatment costs of self-expandable metal stents and plastic stents for management of malignant biliary obstruction
Source: BMC Gastroenterol. 2023 Feb 16;23:41. doi: 10.1186/s12876-023-02668-9 (PMC9933253; doi:10.1186/s12876-023-02668-9)
Supplement: Supplementary file 1 — Additional file 1: Table S1. American Society of Anesthesiologists (ASA) Physical Status Classification; Table S2. Costs used in cost-effectiveness analysis of stent implantation in the management of primary malignant biliary obstruction. [file 12876_2023_2668_MOESM1_ESM.docx]

**Supplementary Table legends**

**Supplementary Table 1.** American Society of Anesthesiologists (ASA) Physical Status Classification.

**Supplementary Table 2.** Costs used in cost-effectiveness analysis of stent implantation in the management of primary malignant biliary obstruction

**Supplementary Table 1.** American Society of Anesthesiologists (ASA) Physical Status Classification.

| **Class** | **Description** |
| --- | --- |
| **ASA I** | Normal healthy patient |
| **ASA II** | Patient with mild systemic disease |
| **ASA III** | Patient with severe systemic disease |
| **ASA IV** | Patient with severe systemic disease that is constant threat to life |
| **ASA V** | Moribund patient who is not expected to survive without the operation |
| **ASA VI** | Patient declared brain-dead patient whose organ are being removed for donor purposes |

**Supplementary Table 2.** Costs used in cost-effectiveness analysis of stent implantation in the management of primary malignant biliary obstruction

| **Cost used for analysis** | **Cost (****€)** |
| --- | --- |
| **SEMS** | 540 |
| **Plastic stent** | 32 |
| **PTD with plastic stent** | 350 |
| **PTD with metal stent** | 910 |
| **ERCP** | 320 |
| **EST** | 95 |
| **Cost of hospitalization/days** | 130 |

ERCP, endoscopic retrograde cholangiopancreatography; EST, endoscopic sphincterotomy; PTD, percutaneous biliary drainage; SEMS, self-expandable metal stent.
